# Plasma phosphorylated tau181 and neurodegeneration in Alzheimer’s disease

**DOI:** 10.1002/acn3.51253

**Published:** 2020-11-29

**Authors:** Oskar Hansson, Nicholas Cullen, Henrik Zetterberg, the Alzheimer’s Disease Neuroimaging Initiative, Kaj Blennow, Niklas Mattsson‐Carlgren

**Affiliations:** ^1^ Memory Clinic Skåne University Hospital Lund Sweden; ^2^ Clinical Memory Research Unit Department of Clinical Sciences Lund University Malmö Sweden; ^3^ Wallenberg Centre for Molecular Medicine Lund University Lund Sweden; ^4^ Clinical Neurochemistry Laboratory Sahlgrenska University Hospital Mölndal Sweden; ^5^ Department of Psychiatry and Neurochemistry Institute of Neuroscience and Physiology Sahlgrenska Academy University of Gothenburg Gothenburg Sweden; ^6^ Department of Neurodegenerative Disease UCL Institute of Neurology London United Kingdom; ^7^ UK Dementia Research Institute at UCL London United Kingdom; ^8^ Department of Neurology Skåne University Hospital Lund Sweden

## Abstract

We examined if plasma phosphorylated tau is associated with neurodegeneration in Alzheimer’s disease. We investigated 372 cognitively unimpaired participants, 554 mild cognitive impairment patients, and 141 Alzheimer’s disease dementia patients. Tau phosphorylated at threonine 181, regional cortical thickness (using magnetic resonance imaging) and hypometabolism (using fluorodeoxyglucose positron emission tomography) were measured longitudinally. High plasma tau was associated with hypometabolism and cortical atrophy at baseline and over time, and longitudinally increased tau was associated with accelerated atrophy, but these associations were only observed in A*β*‐positive participants. Plasma phosphorylated tau may identify and track processes linked to neurodegeneration in Alzheimer’s disease.

## Introduction

Plasma P‐tau181 is increased in Alzheimer’s disease (AD), and correlates with brain deposition of aggregated *β*‐amyloid (A*β*) and tau, the core AD hallmarks.^1^, ^2^, ^3^ Plasma P‐tau181 may potentially be used as a noninvasive proxy for tau pathology linked to neurodegeneration,^2^, ^4^ but longitudinal plasma P‐tau181 data have not been examined. To better understand the performance of plasma P‐tau181 to monitor tau pathology and subsequent neurodegeneration in AD, we need truly longitudinal analyses that incorporate change of both neuroimaging measures and P‐tau181. We tested associations between longitudinal plasma P‐tau181 and imaging measures for hypometabolism, using fluorodeoxyglucose positron emission tomography (FDG‐PET) and for cortical atrophy, using magnetic resonance imaging (MRI), in a large cohort of cognitively unimpaired (CU) individuals, patients with mild cognitive impairment (MCI) and AD dementia patients. We tested the hypothesis that increased plasma P‐tau181 was associated with signs of neurodegeneration, both at baseline and over time. Since plasma P‐tau181 is strongly linked to AD,^1^, ^2^, ^3^ we further hypothesized that these associations would only be seen in individuals who were on the AD trajectory (as indicated by a positive A*β* PET scan).

## Methods

### Study participants

Data were obtained from the Alzheimer’s Disease Neuroimaging Initiative (ADNI) database (adni.loni.usc.edu). The ADNI was launched in 2003 as a public‐private partnership, led by Principal Investigator Michael W. Weiner, MD. For up‐to‐date information, see www.adni‐info.org. We used data accessed at the ADNI database on 2020/06/25. We included all CU controls, MCI and AD dementia patients with plasma P‐tau181 and at least one available MRI or FDG‐PET scan. Inclusion and exclusion criteria have been described before.^5^ In sum, CU participants had Mini Mental State Examination (MMSE) score ≥ 24, and Clinical Dementia Rating (CDR) score 0. MCI participants had MMSE score ≥ 24, objective memory loss tested by delayed recall of the Wechsler Memory Scale Logical Memory II, CDR 0.5, preserved activities of daily living, and absence of dementia. AD dementia patients fulfilled the National Institute of Neurological and Communicative Disorders and Stroke and the Alzheimer's Disease and Related Disorders Association (NINCDS‐ADRDA) criteria for probable AD,^6^ had MMSE 20–26 and CDR 0.5‐1.0. The study data and samples were collected from 2005/10/24 to 2019/07/17. Ethical approval was given by the local ethical committees of all involved sites. All participants gave written informed consent.

### Biomarker and imaging measurements

Plasma samples were taken annually. P‐tau181 was analyzed on a Single molecule array (Simoa) HD‐X Analyzer (Quanterix, Billerica, MA), using an in‐house assay developed in the Clinical Neurochemistry Laboratory, University of Gothenburg, Sweden.^2^ In the P‐tau181 data file (UGOTPTAU181_06_18_20.csv at the ADNI database), we noted 33 outlying data points (P‐tau181‐concentrations more than three standard deviations above the mean, 63.3 ng/L) out of *N* = 3758 observations. We excluded those outliers from all analyses.

Structural brain images were acquired using 3 Tesla MRI scanners with T1‐weighted MRI scans using a sagittal volumetric magnetization prepared rapid gradient echo (MP‐RAGE) sequence. MRI scans were done at baseline, 3 months and 6 months, and thereafter annually. FreeSurfer (v5.1) was used for quantification of regional thickness and volumes according to the 2010 Desikan‐Killany atlas.^7^ We used cortical thickness for a meta‐region of interest (“temporal composite”) involving entorhinal, inferior temporal, middle temporal, and fusiform cortex.^8^ FDG‐PET scans were acquired annually. An FDG composite score was calculated as the average uptake in left and right angular, temporal, and posterior cingulate regions.^9^ 18F‐Florbetapir PET brain scans for A*β* deposition were acquired at baseline according to a previously described protocol,^9^ using a cortical summary‐ROI consisting of frontal, anterior/posterior cingulate, lateral parietal, lateral temporal brain regions and with whole cerebellum as reference. A*β* positivity was defined as 18F‐florbetapir PET > 1.11 SUVR.^9^

### Statistical analyses

We determined associations between continuous plasma P‐tau181 and neuroimaging measures, using all available paired longitudinal data, in linear mixed‐effects (LME) models with P‐tau181 as predictor and neuroimaging measures as response. We next tested if baseline plasma P‐tau181 predicted change in neuroimaging measures. To use all available data for the imaging measures, we extracted slopes of change for temporal cortical thickness and FDG‐PET in separate linear regression models (without requiring paired P‐tau181 data). We then used neuroimaging slopes as response variables in linear regression models with continuous P‐tau181 as the predictor. We also tested if longitudinal change in P‐tau181 correlated with longitudinal change in neuroimaging measures. For this, we extracted slopes for P‐tau181 in separate linear regression models (without requiring paired imaging data). We used those slopes as predictors in linear regression models with a change in neuroimaging measures as outcomes. All these models were adjusted for age, sex, diagnostic group, *APOE* *ε*4 status (positive = at least one *ε*4 allele, or negative = no *ε*4 alleles), A*β* status, and the interaction between A*β* status and P‐tau181, and (for slope data) lag between first imaging scan and first P‐tau181 sampling. For the final analyses, we used binary P‐tau181 status. We defined a P‐tau181 cut‐point using two‐component mixture modeling of the P‐tau181 data in A*β*‐negative CU, at the mean concentration plus two standard deviations of the lower component. We divided the data in four groups by combinations of positivity and negativity on P‐tau181 and A*β* PET. We used this factor as the predictor of baseline and longitudinal neuroimaging measures in linear regression models, adjusted for age, sex, diagnostic group, and (for tests of slopes) lag between the first imaging scan and first P‐tau181 measure. We also performed sensitivity analyses within diagnostic subgroups and in a restricted dataset with paired MRI and FDG‐PET imaging data. All statistical analyses were done in R (v 4.0.0). Significance was determined at *P* < 0.05.

## Results

Demographics are shown in Table 1.

**Table 1:** Demographics.

	CU	MCI	AD dementia
N	372	554	141
Sex (M/F)	196/176	241/313	59/82
Age (y)	73.6 (5.8)	71.9 (7.4)	74.4 (8.2)
Education (y)	16.6 (2.6)	16.1 (2.7)	15.7 (2.7)
*APOE* *ε*4 (−−/− +/++)	267/98/7	290/209/55	46/66/29
Baeline A*β*‐status (−/+)	245/127	243/311	16/125
Baseline P‐tau181 (ng/L)	15.2 (8.6)	18.4 (10.5)	22.8 (8.5)
Baseline temporal cortical thickness (mm)	2.82 (0.15)	2.76 (0.19)	2.53 (0.22)
Baseline FDG‐PET (SUVR)	1.31 (0.11)	1.25 (0.14)	1.07 (0.15)
Nr of P‐tau181 measures (median [IQR])	3 (2–4)	4 (3–5)	2 (1–2)
Duration from first to last P‐tau181 measure (y) (median [IQR])	2.05 (1.98–3.97)	3.01 (2.00–3.99)	0.99 (0.00–1.05)
Nr of MRI scans (median [IQR])	5 (4–6)	5 (4–6)	4 (4–4)
Duration from first to last MRI scan (y) (median [IQR])	2.07 (1.98–4.00)	2.12 (1.98–4.00)	1.02 (0.98–1.95)
Nr of FDG‐PET scans (median [IQR])	2 (1–2)	2 (1–2)	1 (1–1)
Duration from first to last FDG‐PET (y) (median [IQR])	1.95 (0.00–2.03)	1.99 (0.00–3.97)	0.00 (0.00–0.00)

Continuous data are mean (standard deviation).

### Continuous plasma P‐tau181 and temporal cortical thickness

**Figure 1:**
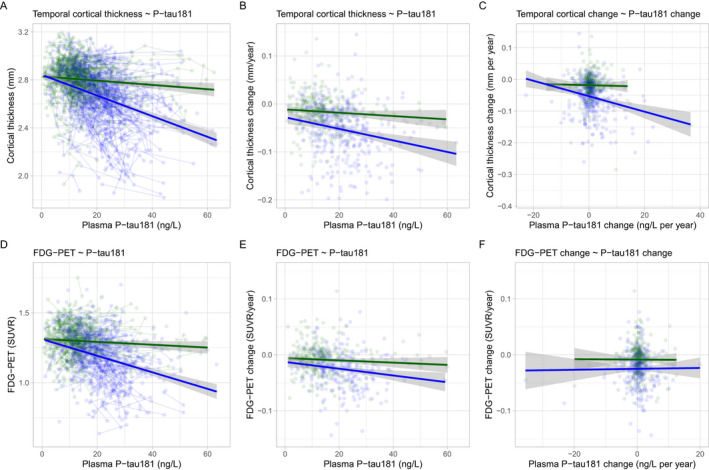
Plasma P‐tau181 and imaging measures of neurodegeneration. Associations between temporal cortical thickness (panels A–C) and fluorodeoxyglucose PET (panels D–F) with plasma P‐tau181 levels, testing associations with all available paired data (panels A and D), associations between slopes of imaging measures and baseline P‐tau181 (panels B and E), and associations between slopes of imaging measures and slopes of P‐tau181 (panels C and F). All models are adjusted for age, sex, diagnostic group, *APOE* *ε*4 status, and A*β* status. Slope models are also adjusted for lag between the first imaging scan and first P‐tau181 measure. The effects in A*β*‐negative subjects are shown in green and effects in A*β*‐positive subjects are shown in blue (for individual subjects and average effect). Panels A and D show results from linear mixed‐effects models, with several data points per individual (individual subjects as a random factor). *P*‐values are extracted from the models for associations in A*β*‐negative and A*β*‐positive subjects separately. *P*‐values are also indicated for the difference in slopes between A*β*‐positive and A*β*‐negative subjects.

Higher plasma P‐tau181 was associated with thinner cortices in A*β*+, but not in A*β*‐ individuals (Fig. 1A; in subgroup analyses, this was found in MCI and AD, but not in CU, Figs. [Link], [Link], [Link]). Higher baseline plasma P‐tau181 was also associated with more rapid decline of cortical thickness in A*β*+, but not in A*β*‐ individuals (Fig. 1B; in subgroup analyses, this was found in MCI, but not in CU or AD, Figs. [Link], [Link], [Link]). Greater slopes of plasma P‐tau181 were associated with more rapid decline of cortical thickness in A*β*+ individuals, but not in A*β*‐ individuals (Fig. 1C; in subgroup analyses, this was found in CU and AD, but not in MCI, Figs. S1, –S3).

### Continuous plasma P‐tau181 and hypometabolism

Higher plasma P‐tau181 was associated with lower FDG‐PET SUVR in A*β*+, but not in A*β*‐ individuals (Fig. 1D; in subgroup analyses, this was found in MCI, but not in CU or AD, Figs. [Link], [Link], [Link]). Higher baseline plasma P‐tau181 was also associated with more rapid decline of FDG‐PET SUVR in A*β*+, but not in A*β*‐ individuals (Fig. 1E; in subgroup analyses, this was found in CU, but not in MCI or AD, Figs. S1, –S3). Slopes of plasma P‐tau181 were not associated with slopes of FDG‐PET SUVR in either A*β*‐ or A*β*+ (Fig. 1F).

### Dichotomous plasma P‐tau181 and neurodegeneration

**Figure 2:**
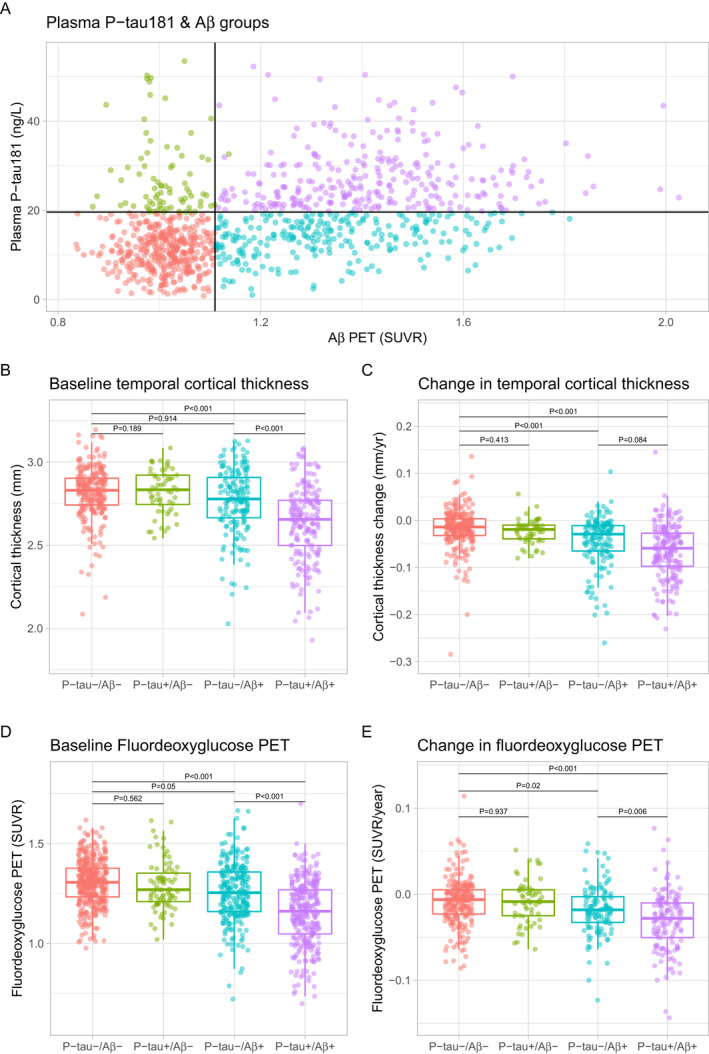
Fluorodeoxyglucose PET and temporal cortical thickness by groups av P‐tau181 and A*β*‐PET positivity. Panel A shows the overall P‐tau181 and A*β* PET data, cut‐points for P‐tau181 (>19.8 ng/L) and A*β* PET (>1.11 SUVR) positivity, and the four groups created by combinations of P‐tau181 and A*β* PET. Panels B–E show differences between groups for temporal cortical thickness and FDG‐PET, at baseline and over time. The groups were compared in linear regression models, adjusted for age, sex, *APOE* *ε*4 status, diagnostic group, and lag between the first imaging scan and first P‐tau181 measure. *P*‐values are extracted for comparisons between P‐tau181−/A*β*‐ (reference) and all other groups, and between P‐tau181‐negative and ‐positive groups within the A*β* groups.

A cut‐point for abnormal baseline plasma P‐tau181 was defined in the A*β*‐negative CU group (P‐tau181 > 19.8 ng/L). The data were divided into four parts based on P‐tau181 and A*β* status (Fig. 2A). P‐tau181+/A*β*+ (*N* = 302) had thinner baseline cortices (in subgroup analyses, this was found in MCI and AD, but not in CU, Figs. [Link], [Link], [Link]), lower baseline FDG‐PET SUVR (in subgroup analyses this was found in CU and MCI, but not in AD, Figs. [Link], [Link], [Link]), and more rapid decline in cortical thickness (but not in subgroup analyses, Figs. [Link], [Link], [Link]) and FDG‐PET SUVR (in subgroup analyses, this was found in MCI, but not in CU or AD, Figs. S4, –S6) than P‐tau181−/A*β*+ (*N* = 261). There were no differences between Ptau181‐/A*β*‐ (N = 425) and P‐tau181+/A*β*‐ (*N* = 79) for any comparison.

### Sensitivity analyses with paired imaging data

In the main analyses, we included all available MRI and FDG‐PET data, as measures of neurodegeneration. Although we did not aim to compare effects on MRI with effects on FDG‐PET, we also did a sensitivity analysis restricted to paired imaging data (from ADNI visits when both MRI and FDG‐PET data were available). This resulted in a smaller dataset, with 206 CU, 376 MCI, and 88 AD dementia patients. Most findings remained significant or borderline significant in this restricted data set (Figs. S7 and S8). In sum, higher plasma P‐tau181 remained associated with thinner cortices, more rapid decline of cortical thickness and lower FDG‐PET SUVR in A*β*+ (but not in A*β*‐) individuals. Greater slopes of plasma P‐tau181 remained associated with more rapid decline of cortical thickness in A*β*+ individuals. P‐tau181+/A*β*+ had thinner baseline cortices and lower baseline FDG‐PET SUVR than P‐tau181−/A*β*+, but the associations with decline in cortical thickness and FDG‐PET SUVR were no longer significant in this subset.

## Discussion

Higher plasma P‐tau181 was associated with reduced brain glucose metabolism, reduced temporal lobe cortical thickness, and more rapid acceleration of hypometabolism and cortical atrophy, indicating worsening of neurodegeneration, but only in those with evidence of AD‐type pathophysiology in the form of a positive A*β* PET scan. Longitudinal increases of plasma P‐tau181 correlated with longitudinal cortical atrophy in A*β* PET‐positive individuals. Taken together, this suggests that plasma P‐tau181 reflects downstream longitudinal neurodegeneration due to AD, linked to either accumulation of paired‐helical filament (PHF) positive neurites surrounding amyloid plaques or to intracellular tangles.

Several studies have found correlations between CSF P‐tau181 and neurodegeneration, although comparisons involving longitudinal CSF data are rare.^10^, ^11^, ^12^ Previous studies have also suggested correlations between plasma P‐tau181 and atrophy, using MRI.^2^, ^4^ Mielke et al. showed that higher plasma P‐tau181 was associated cross sectionally with less cortical thickness in a group of 269 CU individuals, MCI and AD dementia patients, but there were no associations in the individual diagnostic groups.^4^ Karikari et al. showed that plasma P‐tau181 was associated with cross‐sectional and 1‐year longitudinal gray matter atrophy in 88 individuals, including CU, MCI, and AD dementia patients, but again there were no associations within individual diagnostic groups.^2^ Our study is, to our knowledge, the first truly longitudinal study on the topic (using longitudinal data for both imaging measures and plasma P‐tau181). We also found associations when adjusting for clinical diagnosis, which may otherwise confound the relationship between atrophy and biochemical measures.

The longitudinal link between plasma P‐tau181 and signs of atrophy in people with (but not without) biomarker signs of A*β* pathology, suggests that plasma P‐tau181 may be useful as a noninvasive marker to track neurodegeneration in AD. Plasma P‐tau181 also performs well as a diagnostic marker for AD.^1^, ^2^, ^4^ Together, this suggests that measurement of plasma P‐tau181 concentration may be useful in clinical trials (and perhaps in clinical practice) as a noninvasive, affordable way to monitor disease progression. As in previous studies,^1^, ^2^, ^3^ we note high P‐tau181 levels despite a normal A*β* PET scan in a minority of subjects. Since these subjects do not show signs of increased neurodegeneration, this may not indicate a malign condition.

One limitation was that only one cohort was used. ADNI is tailored to represent a clinical trial population, and further studies are needed on more general and diverse populations, where neurodegeneration is more likely to also be impacted by other (non‐AD) processes. The study cohort did, however, include A*β*‐negative MCI and (clinically diagnosed) AD dementia subjects, which are likely to have cognitive impairment due to non‐AD conditions, including depression and cerebrovascular disease.^13^, ^14^ We only included subjects with A*β* data, so that we could stratify the analyses by A*β*‐status, but we did not perform complete ATN classification,^15^ since measures of neurodegeneration were key outcome data, and since we did not have an independent measure of T‐status (tau PET data were lacking in most subjects, and CSF P‐tau181 data were not suitable for this analysis, since it is very closely correlated with plasma P‐tau181, the main predictor in this study^1^, ^3^).

## Author Contributions

NMC and OH conceived the study. NMC and NC performed the statistical analysis. HZ and KB were responsible for biochemical analyses. NMC and OH drafted the initial manuscript. All authors contributed to the revision and editing of the manuscript.

## Conflict of Interest

NC, and NMC have nothing to disclose. OH has acquired research support (for the institution) from Roche, Pfizer, GE Healthcare, Biogen, Eli Lilly and AVID Radiopharmaceuticals. In the past 2 years, he has received consultancy/speaker fees (paid to the institution) from Biogen and Roche. HZ has served at scientific advisory boards for Denali, Roche Diagnostics, Wave, Samumed, Siemens Healthineers, Pinteon Therapeutics and CogRx, has given lectures in symposia sponsored by Fujirebio, Alzecure and Biogen, and is a co‐founder of Brain Biomarker Solutions in Gothenburg AB (BBS), which is a part of the GU Ventures Incubator Program. KB has served as a consultant, at advisory boards, or at data monitoring committees for Abcam, Axon, Biogen, JOMDD/Shimadzu. Julius Clinical, Lilly, MagQu, Novartis, Roche Diagnostics, and Siemens Healthineers, and is a co‐founder of Brain Biomarker Solutions in Gothenburg AB (BBS), which is a part of the GU Ventures Incubator Program.

## Supporting information


**Figure S1.** Plasma P‐tau181 and imaging measures of neurodegeneration in CU subjects.

**Figure S2.** Plasma P‐tau181 and imaging measures of neurodegeneration in MCI subjects.

**Figure S3.** Plasma P‐tau181 and imaging measures of neurodegeneration in AD dementia subjects.

**Figure S4**. Fluorodeoxyglucose PET and temporal cortical thickness by groups av P‐tau181 and A*β*‐PET positivity in CU subjects.

**Figure S5.** Fluorodeoxyglucose PET and temporal cortical thickness by groups av P‐tau181 and A*β*‐PET positivity in MCI subjects.

**Figure S6.** Fluorodeoxyglucose PET and temporal cortical thickness by groups av P‐tau181 and A*β*‐PET positivity in AD dementia subjects.

**Figure S7.** Sensitivity analyses for plasma P‐tau181 and imaging measures of neurodegeneration.

**Figure S8.** Sensitivity analyses for fluorodeoxyglucose PET and temporal cortical thickness by groups av P‐tau181 and A*β*‐PET positivity.
**Data S1.** Member list for the Alzheimer’s Disease Neuroimaging Initiative.

## Data Availability

Anonymized study data for the primary analyses presented herein are available upon request from any qualified investigator for purposes of replicating reported results.

## References

[1] Janelidze S , Mattsson N , Palmqvist S , et al. Plasma P‐tau181 in Alzheimer’s disease: relationship to other biomarkers, differential diagnosis, neuropathology and longitudinal progression to Alzheimer’s dementia. Nat Med 2020;26:379–386. 32123385 10.1038/s41591-020-0755-1

[2] Karikari TK , Pascoal TA , Ashton NJ , et al. Blood phosphorylated tau 181 as a biomarker for Alzheimer’s disease: a diagnostic performance and prediction modelling study using data from four prospective cohorts. Lancet Neurol 2020;19:422–433. 32333900 10.1016/S1474-4422(20)30071-5

[3] Thijssen EH , La Joie R , Wolf A , et al. Diagnostic value of plasma phosphorylated tau181 in Alzheimer’s disease and frontotemporal lobar degeneration. Nat Med 2020;26:387–397. 32123386 10.1038/s41591-020-0762-2 PMC7101073

[4] Mielke MM , Hagen CE , Xu J , et al. Plasma phospho‐tau181 increases with Alzheimer’s disease clinical severity and is associated with tau‐ and amyloid‐positron emission tomography. Alzheimers Dement 2018;14:989–997. 29626426 10.1016/j.jalz.2018.02.013 PMC6097897

[5] Petersen RC , Aisen PS , Beckett LA , et al. Alzheimer’s Disease Neuroimaging Initiative (ADNI): clinical characterization. Neurology 2010;74:201–209. 20042704 10.1212/WNL.0b013e3181cb3e25 PMC2809036

[6] McKhann G , Drachman D , Folstein M , et al. Clinical diagnosis of Alzheimer’s disease: report of the NINCDS‐ADRDA Work Group under the auspices of Department of Health and Human Services Task Force on Alzheimer’s Disease. Neurology 1984;34:939–944. 6610841 10.1212/wnl.34.7.939

[7] Jack CR Jr , Bernstein MA , Fox NC , et al. The Alzheimer’s Disease Neuroimaging Initiative (ADNI): MRI methods. J Magn Reson Imaging 2008;27:685–691. 18302232 10.1002/jmri.21049 PMC2544629

[8] Jack CR , Wiste HJ , Weigand SD , et al. Different definitions of neurodegeneration produce similar amyloid/neurodegeneration biomarker group findings. Brain 2015;138:3747–3759. 26428666 10.1093/brain/awv283 PMC4655341

[9] Landau SM , Mintun MA , Joshi AD , et al. Amyloid deposition, hypometabolism, and longitudinal cognitive decline. Ann Neurol 2012;72:578–586. 23109153 10.1002/ana.23650 PMC3786871

[10] Falcon C , Tucholka A , Monté‐Rubio GC , et al. Longitudinal structural cerebral changes related to core CSF biomarkers in preclinical Alzheimer’s disease: a study of two independent datasets. Neuroimage Clin 2018;19:190–201. 30023169 10.1016/j.nicl.2018.04.016 PMC6050455

[11] Tarawneh R , Head D , Allison S , et al. Cerebrospinal fluid markers of neurodegeneration and rates of brain atrophy in early Alzheimer disease. JAMA Neurol 2015;72:656–665. 25867677 10.1001/jamaneurol.2015.0202 PMC4551490

[12] Wang L , Fagan AM , Shah AR , et al. Cerebrospinal fluid proteins predict longitudinal hippocampal degeneration in early‐stage dementia of the Alzheimer type. Alzheimer Dis Assoc Disord 2012;26:314–321. 22156755 10.1097/WAD.0b013e31823c0cf4 PMC3309103

[13] Nettiksimmons J , DeCarli C , Landau S , Beckett L . Biological heterogeneity in ADNI amnestic MCI. Alzheimers Dement 2014;10:511–521.e1. 24418061 10.1016/j.jalz.2013.09.003 PMC4092059

[14] Landau SM , Horng A , Fero A , Jagust WJ . Amyloid negativity in patients with clinically diagnosed Alzheimer disease and MCI. Neurology 2016;86:1377–1385. 26968515 10.1212/WNL.0000000000002576 PMC4831042

[15] Jack CR , Bennett DA , Blennow K , et al. NIA‐AA Research Framework: toward a biological definition of Alzheimer’s disease. Alzheimers Dement 2018;14:535–562. 29653606 10.1016/j.jalz.2018.02.018 PMC5958625

