# Supplementary material for: Plasma phosphorylated tau181 and neurodegeneration in Alzheimer’s disease
Source: Ann Clin Transl Neurol. 2020 Nov 29;8(1):259–65. doi: 10.1002/acn3.51253 (PMC7818141; doi:10.1002/acn3.51253)
Supplement: Supplementary file 1 — Figure S1. Plasma P‐tau181 and imaging measures of neurodegeneration in CU subjects. [file ACN3-8-259-s001.pdf]

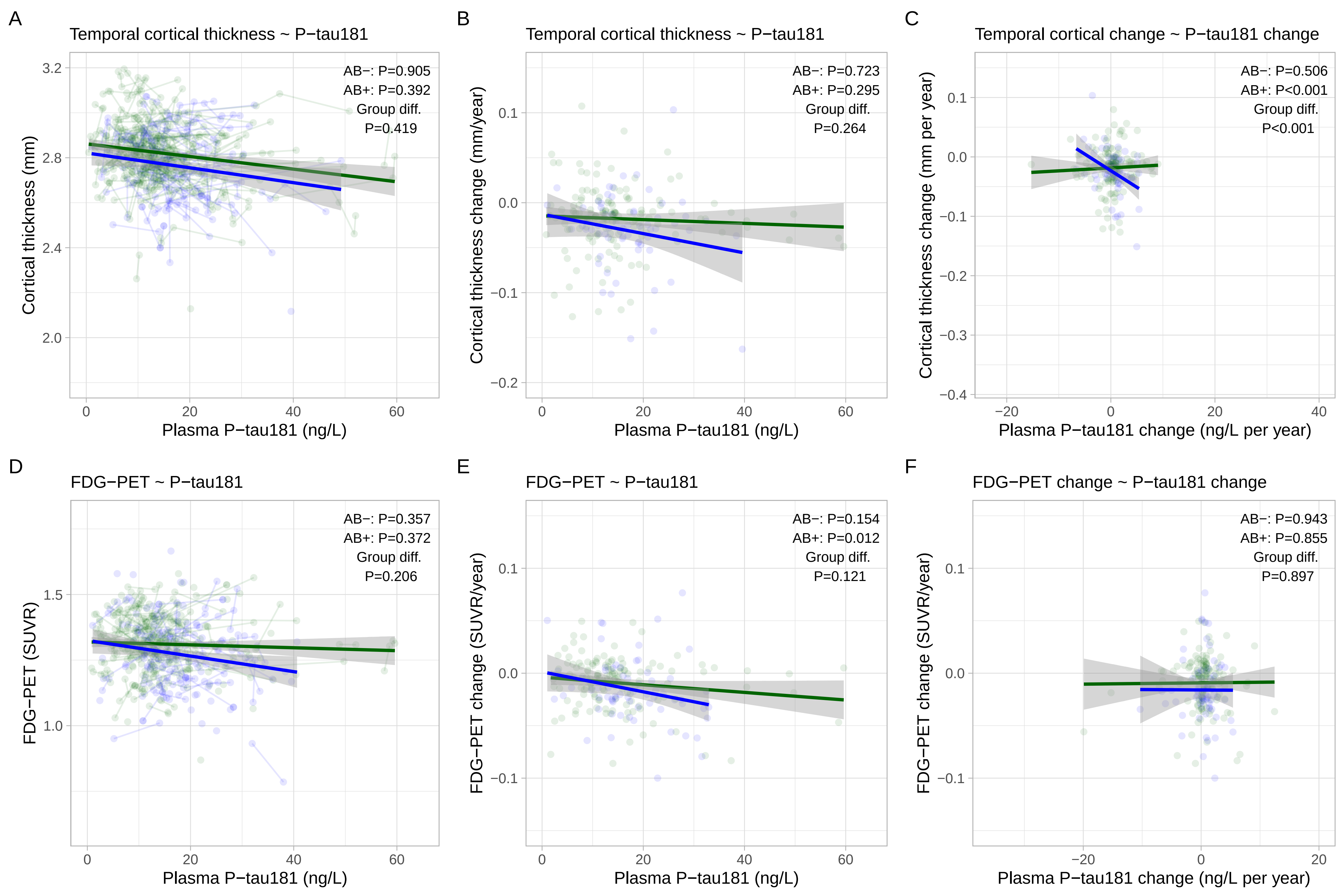
A
Temporal cortical thickness ~ P−tau181
AB−: P=0.905
AB+: P=0.392
Group diff.
P=0.419
Cortical thickness (mm)
Plasma P−tau181 (ng/L)
B
Temporal cortical thickness ~ P−tau181
AB−: P=0.723
AB+: P=0.295
Group diff.
P=0.264
Cortical thickness change (mm/year)
Plasma P−tau181 (ng/L)
C
Temporal cortical change ~ P−tau181 change
AB−: P=0.506
AB+: P<0.001
Group diff.
P<0.001
Cortical thickness change (mm per year)
Plasma P−tau181 change (ng/L per year)
D
FDG−PET ~ P−tau181
AB−: P=0.357
AB+: P=0.372
Group diff.
P=0.206
FDG−PET (SUVR)
Plasma P−tau181 (ng/L)
E
FDG−PET ~ P−tau181
AB−: P=0.154
AB+: P=0.012
Group diff.
P=0.121
FDG−PET change (SUVR/year)
Plasma P−tau181 (ng/L)
F
FDG−PET change ~ P−tau181 change
AB−: P=0.943
AB+: P=0.855
Group diff.
P=0.897
FDG−PET change (SUVR/year)
Plasma P−tau181 change (ng/L per year)
