# Supplementary material for: Plasma phosphorylated tau181 and neurodegeneration in Alzheimer’s disease
Source: Ann Clin Transl Neurol. 2020 Nov 29;8(1):259–65. doi: 10.1002/acn3.51253 (PMC7818141; doi:10.1002/acn3.51253)
Supplement: Supplementary file 2 — Figure S2. Plasma P‐tau181 and imaging measures of neurodegeneration in MCI subjects. [file ACN3-8-259-s002.pdf]

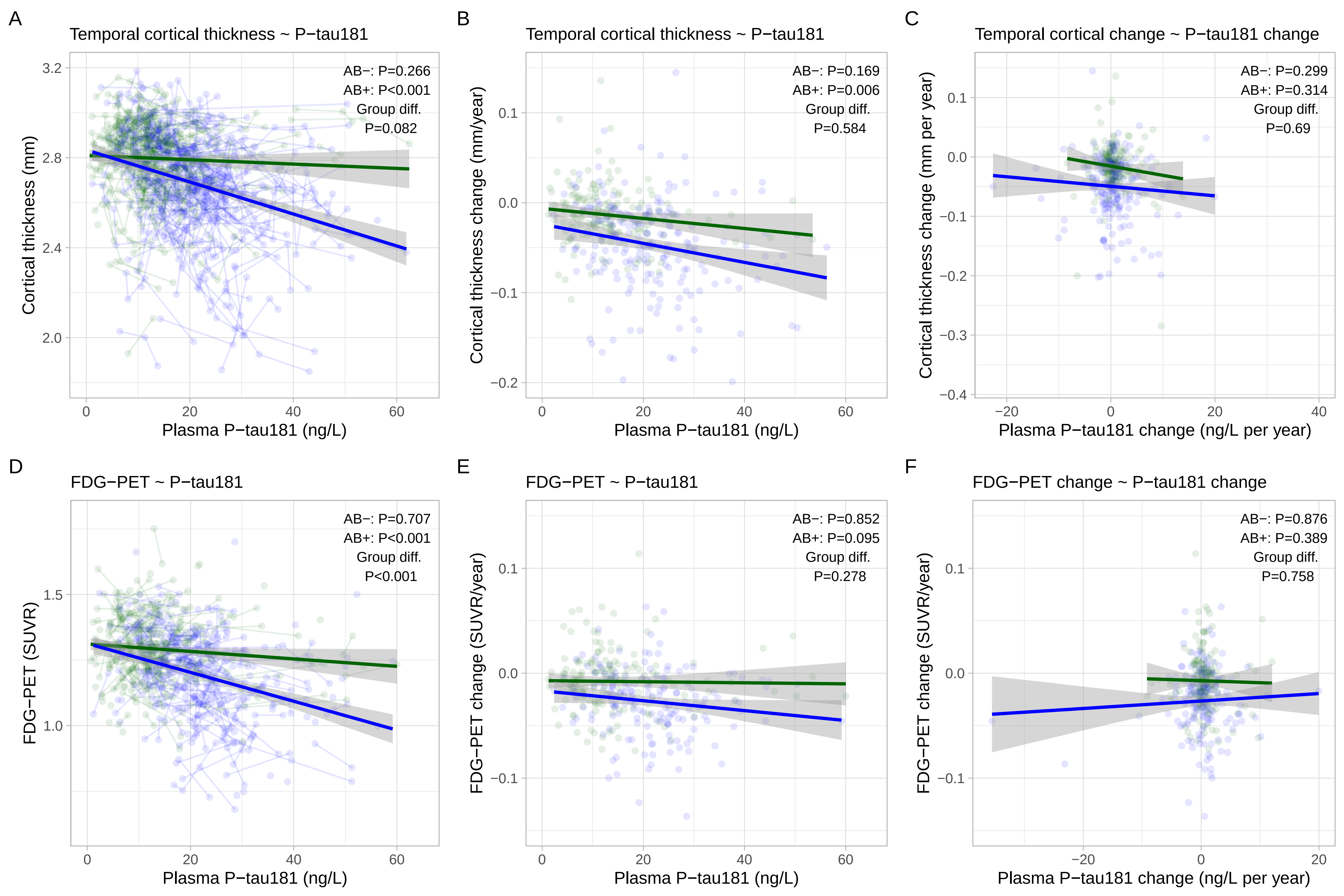
A
Temporal cortical thickness ~ P−tau181
AB−: P=0.266
AB+: P<0.001
Group diff.
P=0.082
Cortical thickness (mm)
Plasma P−tau181 (ng/L)
B
Temporal cortical thickness ~ P−tau181
AB−: P=0.169
AB+: P=0.006
Group diff.
P=0.584
Cortical thickness change (mm/year)
Plasma P−tau181 (ng/L)
C
Temporal cortical change ~ P−tau181 change
AB−: P=0.299
AB+: P=0.314
Group diff.
P=0.69
Cortical thickness change (mm per year)
Plasma P−tau181 change (ng/L per year)
D
FDG−PET ~ P−tau181
AB−: P=0.707
AB+: P<0.001
Group diff.
P<0.001
FDG−PET (SUVR)
Plasma P−tau181 (ng/L)
E
FDG−PET ~ P−tau181
AB−: P=0.852
AB+: P=0.095
Group diff.
P=0.278
FDG−PET change (SUVR/year)
Plasma P−tau181 (ng/L)
F
FDG−PET change ~ P−tau181 change
AB−: P=0.876
AB+: P=0.389
Group diff.
P=0.758
FDG−PET change (SUVR/year)
Plasma P−tau181 change (ng/L per year)
