# Supplementary material for: Plasma phosphorylated tau181 and neurodegeneration in Alzheimer’s disease
Source: Ann Clin Transl Neurol. 2020 Nov 29;8(1):259–65. doi: 10.1002/acn3.51253 (PMC7818141; doi:10.1002/acn3.51253)
Supplement: Supplementary file 3 — Figure S3. Plasma P‐tau181 and imaging measures of neurodegeneration in AD dementia subjects. [file ACN3-8-259-s003.pdf]

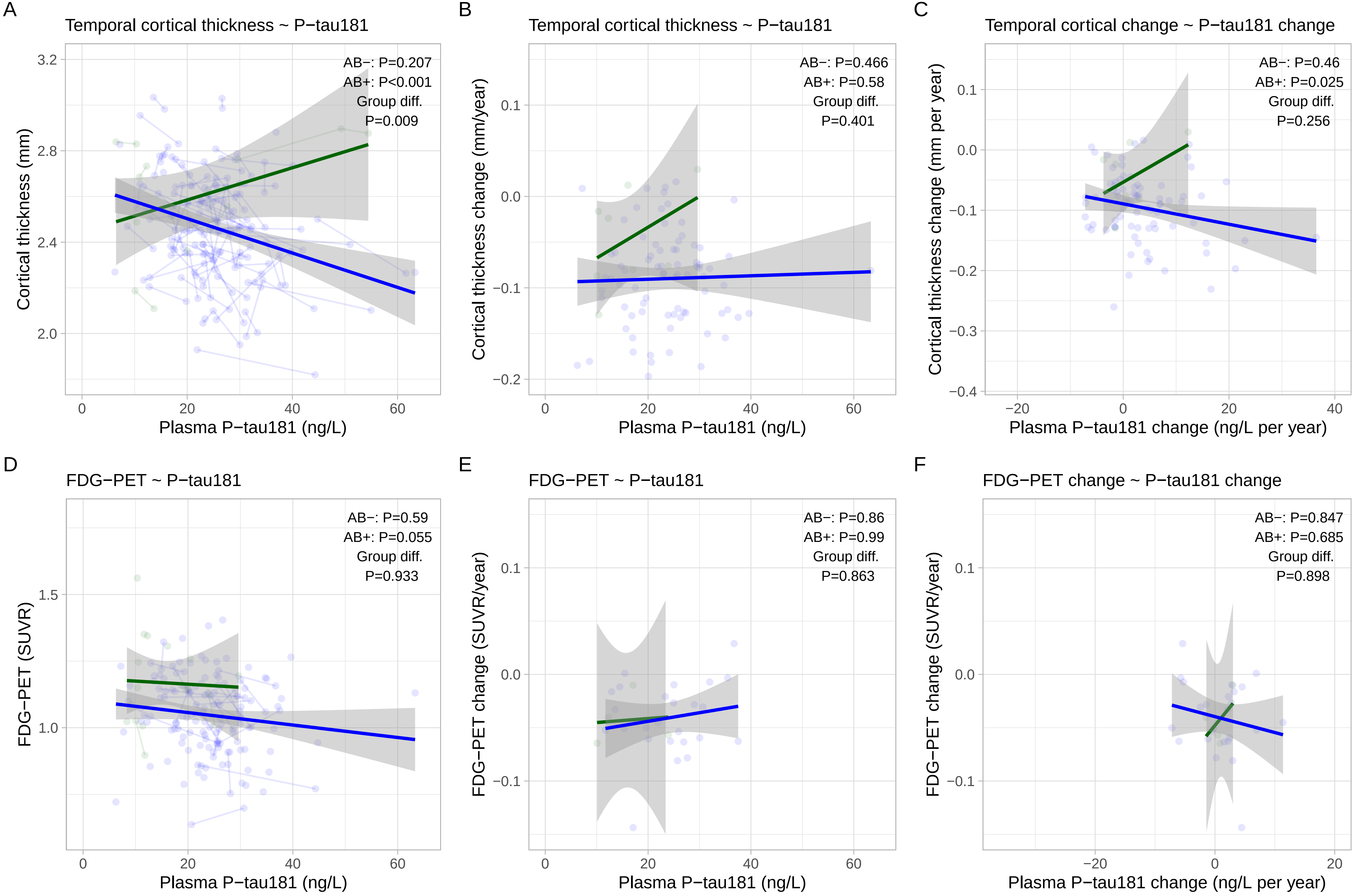

A
Temporal cortical thickness ~ P−tau181
AB−: P=0.207
AB+: P<0.001
Group diff.
P=0.009
Cortical thickness (mm)
3.2
2.8
2.4
2.0
0
20
40
60
Plasma P−tau181 (ng/L)
B
Temporal cortical thickness ~ P−tau181
AB−: P=0.466
AB+: P=0.58
Group diff.
P=0.401
Cortical thickness change (mm/year)
0.1
0.0
−0.1
−0.2
0
20
40
60
Plasma P−tau181 (ng/L)
C
Temporal cortical change ~ P−tau181 change
AB−: P=0.46
AB+: P=0.025
Group diff.
P=0.256
Cortical thickness change (mm per year)
0.1
0.0
−0.1
−0.2
−0.3
−0.4
−20
0
20
40
Plasma P−tau181 change (ng/L per year)
D
FDG−PET ~ P−tau181
AB−: P=0.59
AB+: P=0.055
Group diff.
P=0.933
FDG−PET (SUVR)
1.5
1.0
0
20
40
60
Plasma P−tau181 (ng/L)
E
FDG−PET ~ P−tau181
AB−: P=0.86
AB+: P=0.99
Group diff.
P=0.863
FDG−PET change (SUVR/year)
0.1
0.0
−0.1
0
20
40
60
Plasma P−tau181 (ng/L)
F
FDG−PET change ~ P−tau181 change
AB−: P=0.847
AB+: P=0.685
Group diff.
P=0.898
FDG−PET change (SUVR/year)
0.1
0.0
−0.1
−20
0
20
Plasma P−tau181 change (ng/L per year)
