# Supplementary material for: Plasma phosphorylated tau181 and neurodegeneration in Alzheimer’s disease
Source: Ann Clin Transl Neurol. 2020 Nov 29;8(1):259–65. doi: 10.1002/acn3.51253 (PMC7818141; doi:10.1002/acn3.51253)
Supplement: Supplementary file 4 — Figure S4. Fluorodeoxyglucose PET and temporal cortical thickness by groups av P‐tau181 and Aβ‐PET positivity in CU subjects. [file ACN3-8-259-s004.pdf]

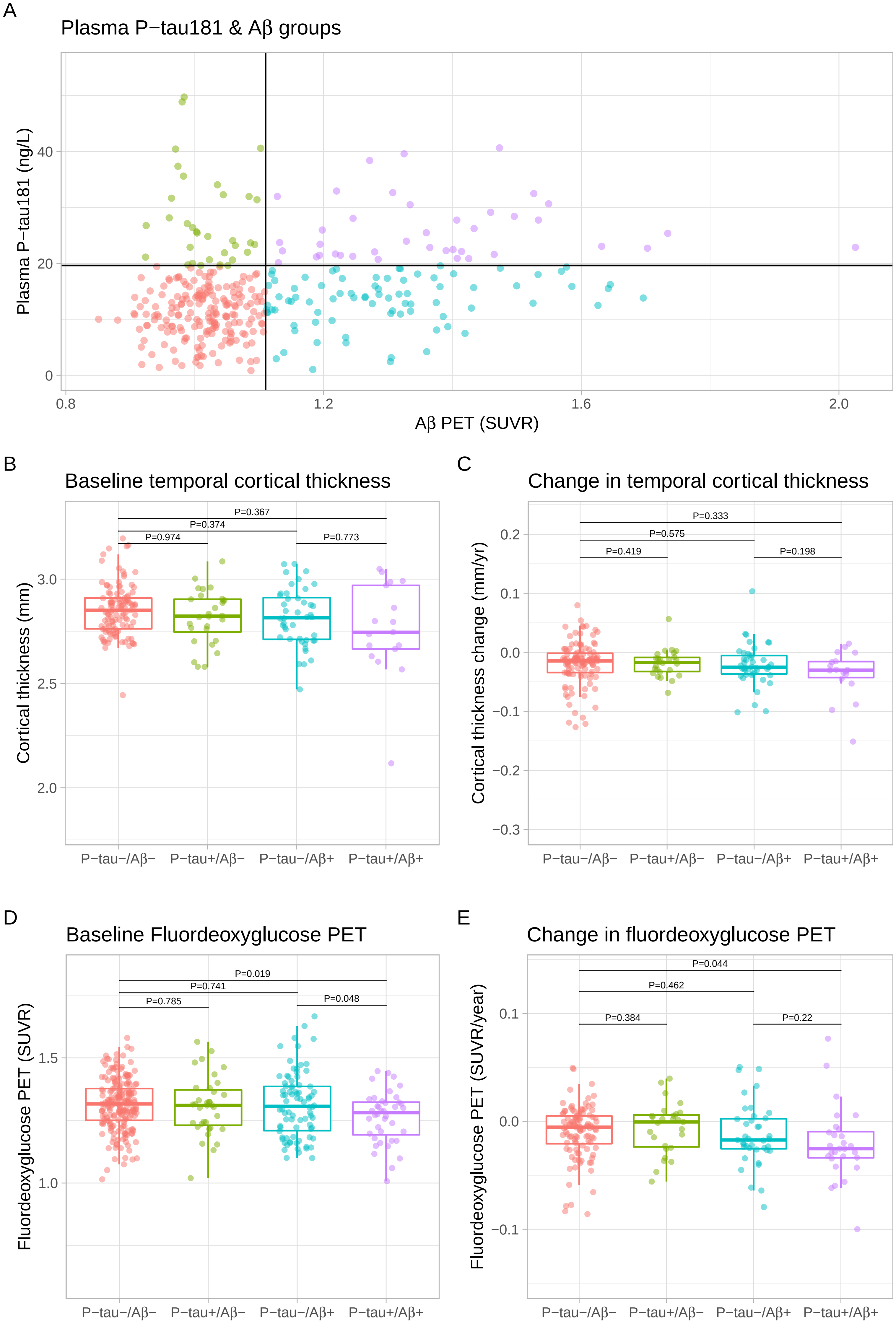

A
Plasma P−tau181 & Aβ groups
Plasma P−tau181 (ng/L)
Aβ PET (SUVR)
B
Baseline temporal cortical thickness
Cortical thickness (mm)
P=0.367
P=0.374
P=0.974
P=0.773
C
Change in temporal cortical thickness
Cortical thickness change (mm/yr)
P=0.333
P=0.575
P=0.419
P=0.198
D
Baseline Fluordeoxyglucose PET
Fluordeoxyglucose PET (SUVR)
P=0.019
P=0.741
P=0.785
P=0.048
E
Change in fluordeoxyglucose PET
Fluordeoxyglucose PET (SUVR/year)
P=0.044
P=0.462
P=0.384
P=0.22
P−tau−/Aβ−
P−tau+/Aβ−
P−tau−/Aβ+
P−tau+/Aβ+
