# Supplementary material for: Plasma phosphorylated tau181 and neurodegeneration in Alzheimer’s disease
Source: Ann Clin Transl Neurol. 2020 Nov 29;8(1):259–65. doi: 10.1002/acn3.51253 (PMC7818141; doi:10.1002/acn3.51253)
Supplement: Supplementary file 5 — Figure S5. Fluorodeoxyglucose PET and temporal cortical thickness by groups av P‐tau181 and Aβ‐PET positivity in MCI subjects. [file ACN3-8-259-s005.pdf]

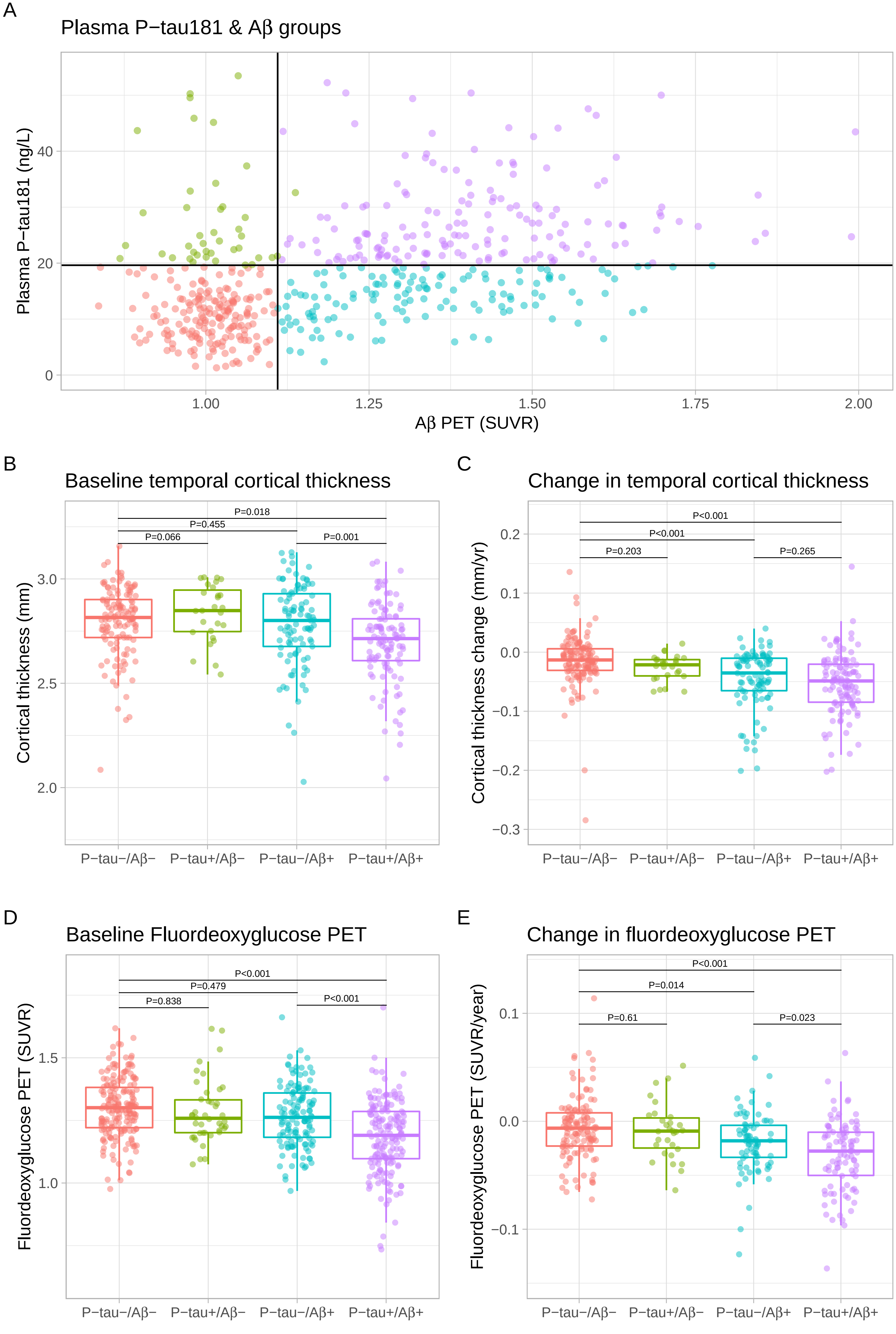

A
Plasma P−tau181 & Aβ groups
Plasma P−tau181 (ng/L)
0
20
40
1.00
1.25
1.50
1.75
2.00
Aβ PET (SUVR)
B
Baseline temporal cortical thickness
Cortical thickness (mm)
2.0
2.5
3.0
P=0.018
P=0.455
P=0.066
P=0.001
P−tau−/Aβ−
P−tau+/Aβ−
P−tau−/Aβ+
P−tau+/Aβ+
C
Change in temporal cortical thickness
Cortical thickness change (mm/yr)
−0.3
−0.2
−0.1
0.0
0.1
0.2
P<0.001
P<0.001
P=0.203
P=0.265
P−tau−/Aβ−
P−tau+/Aβ−
P−tau−/Aβ+
P−tau+/Aβ+
D
Baseline Fluordeoxyglucose PET
Fluordeoxyglucose PET (SUVR)
1.0
1.5
P<0.001
P=0.479
P=0.838
P<0.001
P−tau−/Aβ−
P−tau+/Aβ−
P−tau−/Aβ+
P−tau+/Aβ+
E
Change in fluordeoxyglucose PET
Fluordeoxyglucose PET (SUVR/year)
−0.1
0.0
0.1
P<0.001
P=0.014
P=0.61
P=0.023
P−tau−/Aβ−
P−tau+/Aβ−
P−tau−/Aβ+
P−tau+/Aβ+
