# Supplementary material for: Plasma phosphorylated tau181 and neurodegeneration in Alzheimer’s disease
Source: Ann Clin Transl Neurol. 2020 Nov 29;8(1):259–65. doi: 10.1002/acn3.51253 (PMC7818141; doi:10.1002/acn3.51253)
Supplement: Supplementary file 6 — Figure S6. Fluorodeoxyglucose PET and temporal cortical thickness by groups av P‐tau181 and Aβ‐PET positivity in AD dementia subjects. [file ACN3-8-259-s006.pdf]

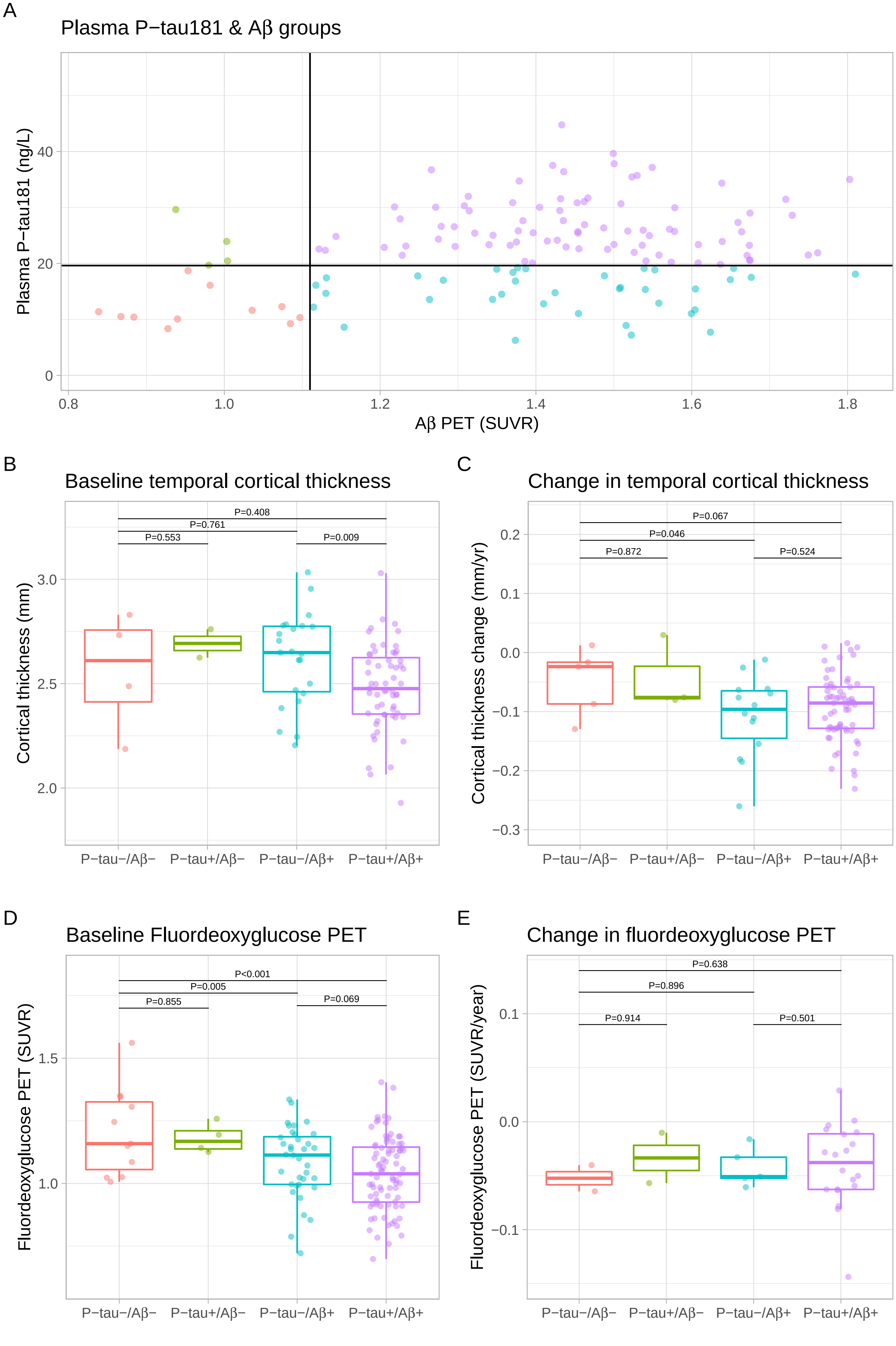
A
Plasma P−tau181 & Aβ groups
Plasma P−tau181 (ng/L)
0
20
40
0.8
1.0
1.2
1.4
1.6
1.8
Aβ PET (SUVR)
B
Baseline temporal cortical thickness
Cortical thickness (mm)
2.0
2.5
3.0
P=0.408
P=0.761
P=0.553
P=0.009
P−tau−/Aβ−
P−tau+/Aβ−
P−tau−/Aβ+
P−tau+/Aβ+
C
Change in temporal cortical thickness
Cortical thickness change (mm/yr)
−0.3
−0.2
−0.1
0.0
0.1
0.2
P=0.067
P=0.046
P=0.872
P=0.524
P−tau−/Aβ−
P−tau+/Aβ−
P−tau−/Aβ+
P−tau+/Aβ+
D
Baseline Fluordeoxyglucose PET
Fluordeoxyglucose PET (SUVR)
1.0
1.5
P<0.001
P=0.005
P=0.855
P=0.069
P−tau−/Aβ−
P−tau+/Aβ−
P−tau−/Aβ+
P−tau+/Aβ+
E
Change in fluordeoxyglucose PET
Fluordeoxyglucose PET (SUVR/year)
−0.1
0.0
0.1
P=0.638
P=0.896
P=0.914
P=0.501
P−tau−/Aβ−
P−tau+/Aβ−
P−tau−/Aβ+
P−tau+/Aβ+
