# Supplementary material for: Plasma phosphorylated tau181 and neurodegeneration in Alzheimer’s disease
Source: Ann Clin Transl Neurol. 2020 Nov 29;8(1):259–65. doi: 10.1002/acn3.51253 (PMC7818141; doi:10.1002/acn3.51253)
Supplement: Supplementary file 8 — Figure S8. Sensitivity analyses for fluorodeoxyglucose PET and temporal cortical thickness by groups av P‐tau181 and Aβ‐PET positivity. Data S1. Member list for the Alzheimer’s Disease Neuroimaging Initiative. [file ACN3-8-259-s008.pdf]

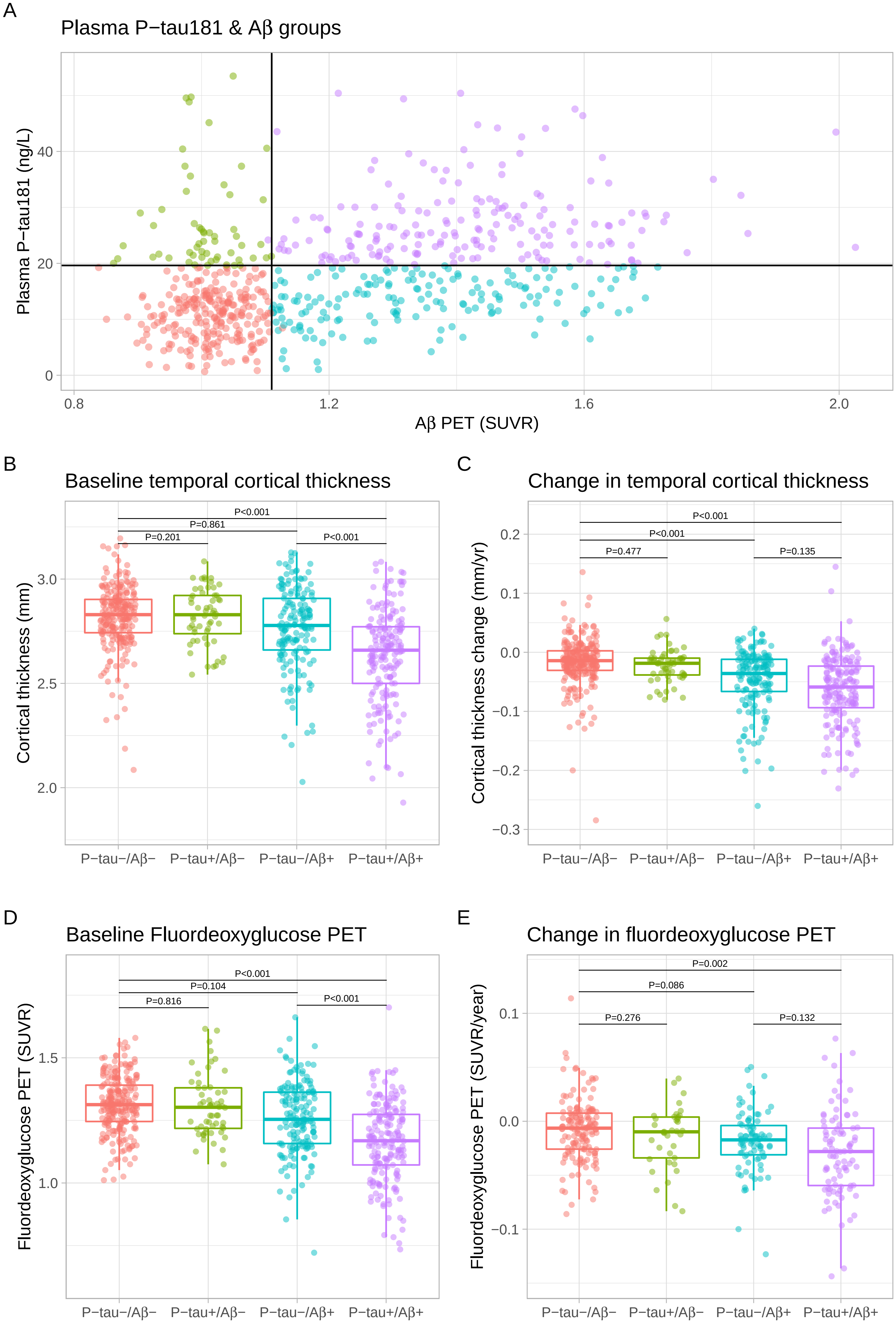

A
Plasma P−tau181 & Aβ groups
Plasma P−tau181 (ng/L)
Aβ PET (SUVR)
0
20
40
0.8
1.2
1.6
2.0
B
Baseline temporal cortical thickness
Cortical thickness (mm)
P=0.201
P=0.861
P<0.001
P<0.001
2.0
2.5
3.0
P−tau−/Aβ−
P−tau+/Aβ−
P−tau−/Aβ+
P−tau+/Aβ+
C
Change in temporal cortical thickness
Cortical thickness change (mm/yr)
P=0.477
P<0.001
P<0.001
P=0.135
−0.3
−0.2
−0.1
0.0
0.1
0.2
P−tau−/Aβ−
P−tau+/Aβ−
P−tau−/Aβ+
P−tau+/Aβ+
D
Baseline Fluordeoxyglucose PET
Fluordeoxyglucose PET (SUVR)
P=0.816
P=0.104
P<0.001
P<0.001
1.0
1.5
P−tau−/Aβ−
P−tau+/Aβ−
P−tau−/Aβ+
P−tau+/Aβ+
E
Change in fluordeoxyglucose PET
Fluordeoxyglucose PET (SUVR/year)
P=0.276
P=0.086
P=0.002
P=0.132
−0.1
0.0
0.1
P−tau−/Aβ−
P−tau+/Aβ−
P−tau−/Aβ+
P−tau+/Aβ+
